# Oscillatory Brain Responses Reflect Anticipation during Comprehension of Speech Acts in Spoken Dialog

**DOI:** 10.3389/fnhum.2018.00034

**Published:** 2018-02-07

**Authors:** Rosa S. Gisladottir, Sara Bögels, Stephen C. Levinson

**Affiliations:** ^1^Max Planck Institute for Psycholinguistics, Nijmegen, Netherlands; ^2^Donders Institute for Brain, Cognition and Behaviour, Radboud University, Nijmegen, Netherlands

**Keywords:** turn-taking, pragmatics, EEG, neuronal oscillations, anticipatory processes, speech acts

## Abstract

Everyday conversation requires listeners to quickly recognize verbal actions, so-called *speech acts*, from the underspecified linguistic code and prepare a relevant response within the tight time constraints of turn-taking. The goal of this study was to determine the time-course of speech act recognition by investigating oscillatory EEG activity during comprehension of spoken dialog. Participants listened to short, spoken dialogs with target utterances that delivered three distinct speech acts (Answers, Declinations, Pre-offers). The targets were identical across conditions at lexico-syntactic and phonetic/prosodic levels but differed in the pragmatic interpretation of the speech act performed. Speech act comprehension was associated with reduced power in the alpha/beta bands just prior to Declination speech acts, relative to Answers and Pre-offers. In addition, we observed reduced power in the theta band during the beginning of Declinations, relative to Answers. Based on the role of alpha and beta desynchronization in anticipatory processes, the results are taken to indicate that anticipation plays a role in speech act recognition. Anticipation of speech acts could be critical for efficient turn-taking, allowing interactants to quickly recognize speech acts and respond within the tight time frame characteristic of conversation. The results show that anticipatory processes can be triggered by the characteristics of the interaction, including the speech act type.

## Introduction

The ability to grasp the function of an utterance in context, i.e., what speech act or verbal action (Searle, 1969; Austin, 1976; Schegloff, 1996, 2007) is being performed, is a critical aspect of successful communication. From the perspective of participants in conversation, speech act recognition is challenging for two reasons. First, it is often the case that an utterance is compatible with multiple speech acts. The statement *I have a car* could be used, for instance, to offer somebody help with moving, to indirectly reject an offer for a ride, or to answer a question. Second, turn-taking in conversation is characterized by tight time constraints, giving participants limited time to recognize the speech act and plan a response. The delay between one speaker’s utterance and another’s reply is most frequently only 200 ms and intervals of 0 ms are common (De Ruiter et al., 2006; Stivers et al., 2009). Given that it takes people at least 600 ms just to produce a one-word utterance (Levelt, 1989; Indefrey and Levelt, 2004), a crucial question is how listeners manage to quickly extract the speech act from the underspecified linguistic code, plan a relevant reply and respond within the 200 ms time frame characteristic of conversation (Levinson, 2013; Bögels et al., 2015; Gisladottir et al., 2015; Levinson and Torreira, 2015).

One proposal is that speech act recognition takes place early in the utterance, thereby allowing listeners to plan a response in time (Levinson, 2013; Gisladottir et al., 2015). Early speech act recognition is in line with models of language and cognition which propose that the brain is “predictive,” “proactive,” or “prospective” and anticipates upcoming events or input based on past experience (see, for instance, Federmeier, 2007; Schacter et al., 2007; Bar, 2009; Van Berkum, 2010; Kutas et al., 2011). A considerably body of behavioral and neuroimaging research has found evidence for anticipation or prediction during language comprehension at several levels, including lexical content (for a review, see Kutas et al., 2011). Conversation analysts have pointed out that the turn-taking system must involve “projection” of the unfolding or upcoming turn, as illustrated with instances where people complete each other’s utterances in conversation (Sacks et al., 1974; Lerner, 1991; Liddicoat, 2004). Supporting this, both adults and children can anticipate turn structure and turn endings, based on a combination of lexical and prosodic cues (De Ruiter et al., 2006; Magyari and de Ruiter, 2012; Casillas and Frank, 2017). However, there is limited research on anticipation at the speech act level, i.e., addressing the question of whether listeners can anticipate the type of action performed in an unfolding or upcoming utterance.

In a recent study using event-related potentials (ERPs) we found partial support for early speech act recognition, reporting speech-act-related ERP effects from 200 ms after speech act onset (Gisladottir, 2015; Gisladottir et al., 2015). The aim of the present study was to further determine the time-course of speech act recognition by investigating oscillatory EEG activity and to shed light on the role of anticipatory processes in speech act recognition. Oscillatory activity contains both phase- and non-phase-locked components (Tallon-Baudry and Bertrand, 1999), while ERPs only contain responses that are phase-locked to stimulus onset and remain after averaging in the time domain (Pfurtscheller and Lopes da Silva, 1999; Bastiaansen et al., 2008). By investigating oscillatory activity, we can get a more complete picture of the time-course of speech act recognition and gain a better understanding of the cognitive processes involved in successful conversation. Suppression of EEG oscillations in the *alpha* (8–12 Hz) and *beta* (13–30 Hz) frequency bands have been associated with anticipatory processing, in relation to motor preparation (see, for instance, Pfurtscheller and Lopes da Silva, 1999), expectations during sensory processing (van Ede et al., 2010; Todorovic et al., 2015) and anticipatory attention, i.e., when attention is oriented toward an upcoming stimulus to facilitate its processing (e.g., Bastiaansen and Brunia, 2001; Bastiaansen et al., 2001; Jones et al., 2010; van Ede et al., 2014). In the language domain, a link between beta desynchronization and anticipation was established in a study using conversational turns with predictable vs. non-predictable endings (Magyari et al., 2014). Predictable turns were accompanied by a power decrease in the low beta band (11–18.5 Hz) as early as 1250 ms before the turn ended, which was taken to reflect early anticipation of turn-endings via syntactic, semantic, and temporal processing.

Based on these studies, one may predict that anticipatory processing plays a role in facilitating early speech act recognition, as reflected by lower alpha and/or beta power before and during the beginning of anticipatable speech acts. Speech act recognition may also involve modulations of *gamma* oscillations (30–100 Hz). An increase in gamma band power has been reported in studies on pragmatic phenomena such as irony (Spotorno et al., 2013) and world knowledge violations (Hagoort et al., 2004). More generally, it has been suggested that gamma plays a functional role in sentence-level language comprehension (Hald et al., 2006; Lewis et al., 2016).

To examine the time-course of speech act recognition and the involvement of anticipatory processes we investigated oscillatory brain responses during comprehension of speech acts in short, spoken dialogs. To our knowledge, no prior studies have investigated oscillatory activity during comprehension of spoken speech acts embedded in conversational contexts. The dialogs contain two utterances; a context utterance by one speaker followed by a target reply from another speaker. The target utterances (replies) perform three different target speech acts; *Answers, Declinations*, and *Pre-offers* (see **Table 1**). Importantly, the targets are identical across the three speech act conditions at lexico-syntactic and phonetic/prosodic levels but differ in the pragmatic interpretation of the speech act performed. The experimental materials are the same as those used in a previous ERP study (with the addition of filler dialogs) (Gisladottir et al., 2015). The Declination and Answer conditions contain target utterances that are relatively anticipatable since the context (the first utterance) highly constrains what type of speech act can follow. This is not the case for the Pre-offer condition, in which the context utterance is less constraining (see section “Materials and Design” for a more detailed description of the stimuli). Thus Declinations and Answers are more anticipatable than Pre-offers.

**Table 1:** Examples of stimuli in Dutch (critical conditions and fillers) and English translations.

Critical condition/filler	Features		Context utterance	Target utterance
Answer	Context is highly constraining	Ex1	*Hoe ga je voor het ticket betalen?*	*Ik heb een creditcard.*
	Target utterance is relatively direct		How are you going to pay for the ticket?	I have a credit-card.
	Target utterance ends the sequence	Ex2	*Waar koop je je shampoo?*	*Ik ga naar de Kruidvat.*
			Where do you buy your shampoo?	I go/am going to the Kruidvat [drugstore].
Declination	Context is highly constraining	Ex1	*Ik kan je wat geld lenen voor het ticket.*	*Ik heb een creditcard.*
	Target utterance is indirect		I can lend you money for the ticket.	I have a credit-card.
	Target utterance ends the sequence	Ex2	*Ik kan wel shampoo voor je meenemen?*	*Ik ga naar de Kruidvat.*
			I can bring some shampoo for you?	I go/am going to the Kruidvat [drugstore].
Pre-offer	Context is not highly constraining	Ex1	*Ik heb geen geld om het ticket te betalen.*	*Ik heb een creditcard.*
	Target utterance is indirect		I don’t have any money to pay for the ticket.	I have a credit-card.
	Target utterance starts a new sequence	Ex2	*Mijn shampoo is op.*	*Ik ga naar de Kruidvat.*
			My shampoo is finished.	I go/am going to the Kruidvat [drugstore].
Answer filler	All fillers:		*Wat neem jij mee naar het etentje?*	*Dat weet ik nog niet.*
	First utterance performs the same speech act as the corresponding critical condition		What are you bringing to the dinner?	I don’t know yet.
Declination filler			*Ik kan een kaartje voor je kopen als je wil.*	*Dat zou ik fijn vinden.*
	Second utterance performs a different speech act than the corresponding critical condition		I can buy a ticket for you if you want.	I would like that.
Pre-offer filler			*Ik moet iemand vinden die deze Engelse tekst wil vertalen.*	*Mijn Engels is echt slecht.*
			I need to find somebody who can translate this text in English.	My English is really bad.

Based on the assumption that reduced alpha/beta power reflects anticipatory processes that may play a role in early speech act recognition, we hypothesized that Declinations and Answers would be associated with lower power in the alpha/beta bands just before or during the beginning of the target utterance relative to Pre-offers. As for Pre-offers, we hypothesized that the EEG signal would show evidence of speech act recognition only late in the utterance, reflected by oscillatory power differences at the utterance final word relative to both Answers and Declinations. We speculated that these final-word power differences would involve the gamma frequency range, due to the association between gamma power and pragmatic language comprehension (Hagoort et al., 2004; Spotorno et al., 2013).

## Materials and Methods

### Participants

Forty-seven native speakers of Dutch participated in the experiment (30 women, mean age = 21.2 years, age range 18–27). A complete EEG recording could not be obtained for one participant. An additional participant was removed due to a noisy EEG signal. The remaining dataset contained data from 45 participants. All participants were right handed, had normal or corrected-to-normal vision and no hearing or speech problems. Participants gave written informed consent according to the Declaration of Helsinki prior to the study. Participants received approximately 20 Euros for participating (8 Euros per hour). The study was approved by the Ethische Commissie Gedragswetenschappelijk Onderzoek at Radboud University Nijmegen.

### Materials and Design

The materials were the same as those used by Gisladottir et al. (2015) with the addition of filler dialogs. The experimental dialogs consist of two spoken utterances, a context utterance (by speaker A) followed by a target utterance (speaker B). The manipulation of interest concerns what speech act is being performed in the target utterance; an Answer, Declination or Pre-offer. Importantly, the target utterances are identical across conditions in terms of lexico-semantic content and phonetics/prosody.

The Answer dialogs contain a question – answer sequence (e.g., *How are you going to pay for the ticket? – I HAVE A CREDIT CARD*). Using the terminology developed by research in conversation analysis, this condition consists of an *adjacency pair* (Schegloff, 2007); the first utterance (question) strongly constrains what type of action can follow (an answer).

The Declination dialogs contain an offer, followed by a declination (e.g., *I can lend you money for the ticket. – I HAVE A CREDIT CARD*). As in the Answer dialogs, the context heavily constrains the critical utterance in this condition due to an adjacency pair structure (given an offer, one should expect either an acceptance or declination). The Declinations are, however, more indirect than the Answers, as more inferencing is required to understand the action.

The Pre-offer dialogs consist of a trouble statement, followed by an action that has been called a *pre-offer* in conversation analysis (Schegloff, 1988, 2007) (e.g., *I don’t have any money to pay for the ticket*. – *I HAVE A CREDIT CARD*). Pre-offers are so named because they frequently precede more direct offers. The Pre-offer dialogs do not constitute an adjacency pair since the context utterance can be followed by a large number of speech acts, only one of which is a pre-offer. The utterance *I don’t have any money* could, for instance, be followed by responses such as condolences (*Oh dear, that sucks*), a telling of one’s own experience (*Me neither*), or a suggestion (*Why don’t you ask somebody for a loan?*); a direct offer or a pre-offer are just two possibilities. As a consequence the Pre-offers occur in less constraining action contexts than Answers and Declinations. However, Pre-offers have in common with Declinations that they are more indirect than Answers.

In total there were 126 target utterances, each presented in three different contexts, making up 378 dialogs. To maintain a balance of variety and control in the stimulus materials, half of the target sentences started with “I have” (Dutch *ik heb*), e.g., “I have a credit card.” The other half was more varied and included simple utterances like “I am going to the market” and “My brother is a mechanic.” We varied the length of the utterances to make the stimuli as natural as possible, but constructed the target utterances such that the final word is critical for understanding the propositional content of the utterance (irrespective of speech act level meaning).

Mean context utterance duration was 1844 ms (*SD* 451 ms) and mean target utterance duration was 1175 ms (*SD* 236 ms). The target utterances contained 3–7 words (median: 4 words). The sentences were recorded in a soundproof booth. Four native speakers of Dutch (two male, two female) were instructed to act out the dialogs as naturally as possible in four different pairings. The context sentences were extracted from those recordings, while the target utterances were recorded separately from a list (without context) to prevent the prosody of the critical utterance from biasing one condition over another. The overall sound intensity of the recordings was normalized using the scale intensity command in Praat (Boersma, 2001), with 75 dB SPL as the new average intensity.

Three types of filler dialogs were created to reduce strategic processing of the target speech acts. The first utterance of the fillers contains the same speech act as the context of the corresponding experimental condition, while the second utterance (a full sentence) delivers a speech act different from the target utterance. Thus the filler dialogs corresponding to the Answer condition consisted of a question followed by a “non-answer” (e.g., *What are you bringing to the dinner? – I don’t know yet*). The fillers corresponding to the Declination condition contained an offer – acceptance sequence (e.g., *I can buy a ticket for you if you want* – *I would like that*). The fillers corresponding to the Pre-offer condition involved a trouble statement, followed by a speech act that did not offer a solution to the problem (e.g., *I need to find somebody who can translate this text in English* – *My English is really bad*). In this design, the critical speech acts cannot be predicted based on the context utterances. As an example, an offer was equally likely to be followed by an acceptance (filler) or a declination (experimental condition). None of the fillers and the experimental dialogs contain anomalies of any kind. They resemble natural conversations between friends or relatives and span common discourse topics such as working/studying, doing groceries, and going out.

The stimuli and fillers were pseudo-randomized and balanced across three lists, such that each list contained 126 experimental items and 126 fillers (with equal number of trials across conditions and filler types). Thus each participant heard each critical dialog only once, in one of the three conditions. The recording session, which included a practice with six dialogs, lasted approximately an hour.

### Procedure

**FIGURE 1:**
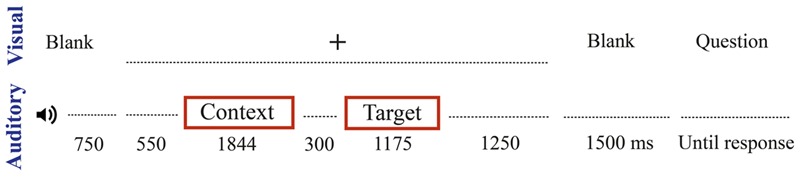
Schematic illustration of the experimental procedure (one trial). Between trials a blank screen was presented for 4000 ms.

Participants received written instructions to listen carefully to the dialogs and try to put themselves in the position of the speakers. The stimuli were presented auditorily through loudspeakers. The paradigm is described in **Figure 1**. Each trial began with a short warning beep and after a 750 ms delay, a fixation cross appeared in the middle of the computer screen. Participants were told to avoid eye blinks and movements during the presentation of the cross, which lasted throughout the entire dialog. The context utterance was played 550 ms after the appearance of the fixation cross. The target utterance was played 300 ms after the offset of the context utterance, such that the no-speech gap between the context and the target turns was 300 ms. The fixation cross stayed on the screen for 1250 ms after the target utterance offset and was followed by a 1500 ms blank screen interval. A comprehension question then appeared on the screen (e.g., *Speaker B gives A the information asked for. – True/Not true*). Participants responded by navigating the screen with a computer mouse and clicking on the answer. Upon answering the comprehension probe, a blank screen appeared for 4000 ms and then the next trial began.

### EEG Recording

The EEG was recorded with 60 active Ag/AgCI electrodes in a cap (actiCAP from Brain Products), referenced to the left mastoid and later re-referenced to the average of the left and right mastoids. Vertical and horizontal eye movements were recorded using four additional electrodes placed above and below the left eye and on the outer canthi. Bipolar EOG was computed. Electrode impedance was kept below 20 KΩ. EEG and EOG recordings were amplified through BrainAmp DC amplifiers (from Brain Products). EEG and EOG data were filtered by a 0.02 Hz high pass and 250 Hz low pass filter with a 10 s time constant and sampled with a frequency of 500 Hz.

### Behavioral Data Analysis

The accuracy data were analyzed with mixed-effects logistic regression using the lme4 package (Bates et al., 2012) in the statistics software R (R Core Team, 2013). Mixed-effects logistic regression is well-suited for the analysis of categorical outcome variables (Jaeger, 2008) such as the question–answer accuracy variable in the current experiment. In particular, it circumvents the limitations of ANOVA in the analysis of categorical variables; when ANOVA is performed on proportions or percentages it leads to problems of interpretability due to confidence intervals that can extend beyond the interpretable values of 0 and 1 and ultimately can yield spurious results (Jaeger, 2008). An additional advantage of mixed-effects logistic regression is that it has greater power than ANOVA to detect true effects and in contrast to ordinary regression it allows the inclusion of participants and items as random effects (Baayen et al., 2008; Jaeger, 2008). The mixed-effects logistic regression analysis of the accuracy data included speech act type as a fixed effect, in addition to random intercepts by participant and item (capturing how participants and items vary in overall accuracy). This was the maximal random effects structure justified by the experimental design and for which convergence was reached, as recommended by Barr et al. (2013).

### Time-Frequency Analysis of Oscillatory Power

To reduce boundary effects in the subsequent time-frequency analysis, the EEG data were first segmented into large epochs: from -1000 ms to 1000 ms relative to the onset of the first word of the target utterance, and from -600 ms to 1700 ms relative to the final word of the target utterance. Then, PCA was used to reduce data dimensionality for each participant to 40 components, which were then subjected to ICA (Oostenveld et al., 2011). These components were inspected visually and removed if they contained only noise and/or artifacts (e.g., caused by eye movements or very noisy electrodes). The average number of removed components was 2.7 (range: 1–8). The remainder of the components was used to recreate the EEG signal. Following this, an automatic procedure was performed to exclude trials exceeding ±75 μV at eye-monitoring sites and ±100 μV at other sites^1^. Data from only 1 participant with less than 22 remaining trials per condition were removed from further analysis (remaining participants *N* = 44). Mean number of trials used for the analysis of the first word in the target utterance was 37.4 in Answers (*SD* 4.2), 37.4 in Pre-offers (*SD* 4.4) and 37.4 in Declinations (*SD* 4.3); mean number of trials used for the analysis of the final word in the target utterance was 36.0 in Answers (*SD* 5.3), 36.0 in Pre-offers (*SD* 5.1) and 36.1 in Declinations (*SD* 6.2). The number of trials left for analysis in the final dataset was 9697 or 87.5%.

To capture both early and late speech act comprehension effects, two critical time-windows of interest in these larger epochs were defined; an *early utterance time-window* from -300 to 600 ms relative to the onset of the first word in the target utterance, and a *final word time-window* from 0 to 1000 ms after onset of the final word in the target utterance. These two time-windows are similar to those used in the previous ERP study (Gisladottir et al., 2015), except that the early utterance time-window here includes the 300 ms interval (silence) before the target utterance to capture pre-stimulus anticipatory effects.

Time-frequency representations (TFRs) of power were computed at the single trial level with a sliding time-window approach using the Matlab toolbox FieldTrip (Oostenveld et al., 2011). For the low-frequency range (2–30 Hz), power was calculated with a Hanning taper, using 400 ms time-windows that were advanced in steps of 10 ms and 1 Hz. Furthermore, two additional control analyses were performed. First, to make sure that effects were not due to phase-locked activity such as onset responses, the same analysis was performed on the raw data per trial from which we subtracted the average ERP of that participant. The results are described briefly in the Section “Results” below. Second, since the time window of 400 ms is relatively short for the lower frequencies (i.e., does not include many cycles), we performed the same analysis with time-windows of 600 ms for the theta range (2–5 Hz) only for the comparisons where we found significant effects in theta in the main analyses. The results are described briefly below. For the higher frequencies (30–90 Hz) a multitaper was used, with 400 ms sliding time-windows, advanced in steps of 10 ms and 2.5 Hz, with frequency smoothing of 5 Hz. Multitapers yield better frequency smoothing which is advantageous for EEG signals above 30 Hz^2^ (for a similar approach combining Hanning and multitapers, see for instance Wang et al., 2012; Nieuwland and Martin, 2017). Since the experimental paradigm does not include a stimulus-free baseline period immediately prior to the critical time-windows, a baseline correction was not used. The non-baselined TFRs of power were averaged over trials for each participant in each condition (Answers, Declinations and Pre-offers).

### Statistical Analysis of TFRs of Power

The participant-level TFRs of power (averaged over trials for each participant in each condition) were submitted to non-parametric cluster-based permutation tests (Maris and Oostenveld, 2007) in Fieldtrip (Oostenveld et al., 2011). This approach has the advantage of offering a straightforward way to deal with the multiple comparisons problem. We first compared all three conditions with ANOVA *F*-tests to see where the three conditions differed overall. Following, two experimental conditions were compared at a time (i.e., Declinations vs. Pre-offers, Answers vs. Pre-offers and Declinations vs. Answers) using *t*-tests. The procedure is as follows. First, an ANOVA (*F*-test) or dependent-samples *t*-test was performed for every channel-frequency-time point in the TFR. Samples that passed a predetermined threshold (*p* < 0.05) were selected and clustered based on adjacency in time, space, and/or frequency. The cluster-level statistics were calculated by taking the sum of all *t*-values or *F*-values within the cluster. The Monte Carlo method was then used to determine the significance of the cluster. A null distribution that assumes no differences between conditions was created by randomly assigning participant averages to one of the two conditions. This procedure was repeated 1000 times and cluster-level statistics were computed for each randomization (as above). Finally, the observed cluster-level test statistics are compared to the null distribution; the Monte Carlo estimate of the *p*-value is the proportion of random partitions resulting in a larger test statistic than the observed cluster. The two experimental conditions were then considered significantly different if the *p*-value was smaller than the critical alpha value, which was set at 0.05 and corrected for a two-tailed test, effectively 0.025.

## Results

### Behavioral Data

Behavioral responses from all participants included in the time-frequency analysis were analyzed (*N* = 44). Overall mean accuracy in the comprehension question was 92.7% (*SD* 26.1%). Participants correctly answered the question for 97.4% of Answers (*SD* 15.9%), 95.5% of Declinations (*SD* 20.8%) and 85.2% of Pre-offers (*SD* 35.5%). The mixed-effects logistic regression model of the accuracy data included speech act type as a fixed effect, in addition to random intercepts by participant and item. The model indicated that the comprehension question was answered less accurately for Pre-offers and Declinations than for Answers (Pre-offers Estimate: -2.24, *SE*: 0.17, *z* = -13.12, *p* < 0.001; Declinations Estimate: -0.71, *SE*: 0.19, *z* = -3.74, *p* < 0.001). However, responses to the Declination questions were more accurate than to Pre-offers (Estimate: 1.54, *SE*: 0.14, *z* = 11.12, *p* < 0.001).

### EEG Results

#### Early Utterance Time-Window

The cluster-based ANOVA approach comparing the three conditions in the lower frequencies (2–30 Hz) showed two marginally significant clusters (*p* = 0.069; *p* = 0.089; see Supplementary Figure 1), one in the alpha/low beta band (11–18 Hz) from about -200 to 0 ms and one in the theta band (2–8 Hz) approximately from -50 to 200 ms. In the cluster-based ANOVA analysis on the lower frequencies with average ERPs subtracted, one significant cluster was found (*p* = 0.008) encompassing both marginal clusters described above. Given the latter result and the fact that we had hypotheses about differences between specific pairs of conditions, we proceeded to look at the pairwise comparisons. The additional cluster-based ANOVA for theta frequencies using larger time-windows (600 ms) did not show any significant clusters (*p* > 0.19).

**FIGURE 2:**
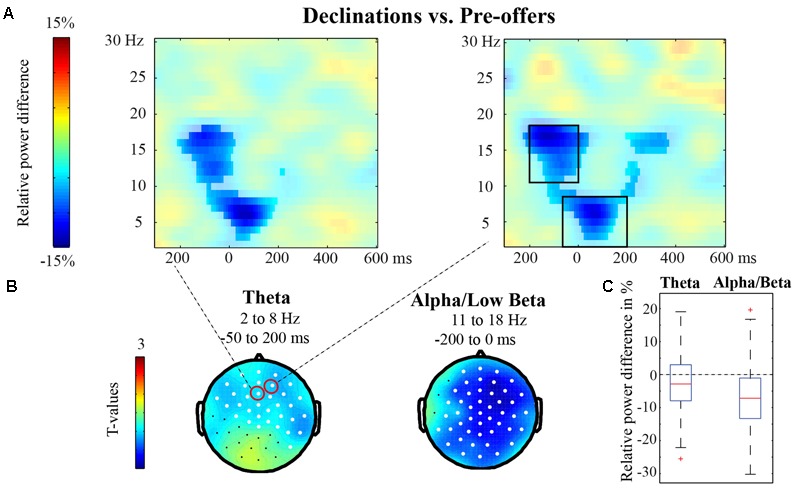
Early utterance time-window: Declinations vs. Pre-offers. **(A)** Relative power differences between Declinations and Pre-offers (in transparent colors) at two representative frontal sites with the significant cluster overlaid in opaque colors. For location of the sites, see circles in **(B)**. **(B)** Topography of the effects (in *t*-values), with channels that showed a significant difference between the conditions highlighted in white. **(C)** Box-plots showing the spread of the relative power differences over participants in the right electrode displayed in **(A)** and the time- and frequency-ranges indicated with black boxes in **(A)** and with text in **(B)**.

We had hypothesized that Declinations and Answers would be associated with lower power in the alpha/beta bands just before or during the beginning of the target utterance, relative to Pre-offers. Thus we first investigated whether there were differences in oscillatory power between Declinations and Pre-offers in the early utterance time-window, i.e., from -300 to 600 ms after target utterance onset. The non-parametric cluster-based permutation test for the low-frequency range (2–30 Hz) revealed one significant cluster in the comparison between Declinations and Pre-offers (*p* < 0.001), which was negative. The results are shown in **Figure 2A**, which presents the relative power differences between Declinations and Pre-offers (divided by their sum) at two representative channels with the significant cluster overlaid in opaque colors. Lower power was observed in Declinations relative to Pre-offers mainly in the alpha/low beta band (11–18 Hz) from -200 to 0 ms and in the theta band (2–8 Hz) approximately from -50 to 200 ms. These effects correspond to the power differences observed in the overall ANOVA for the three speech act conditions. In the analyses with subtracted average ERPs, results were almost identical (one negative cluster with *p* < 0.001; see Supplementary Figure 2). The theta band results were, however, not replicated in the separate theta analysis (with 600 ms windows; *p* > 0.3). The statistical difference in alpha/low beta band is in line with our predictions. **Figure 2B** shows the topography of the effects (in *t*-values), highlighting channels that showed a significant difference between the conditions at some point in the relevant time-frequency window. As seen in **Figure 2B**, the effect was widespread in the alpha/beta band but most prominent at anterior sites in theta. **Figure 2C** shows the spread of the relative power differences over participants at the channel on the right in **Figure 2A**, in the time- and frequency-ranges indicated with black boxes in **Figure 2A**.

We then compared Answers and Pre-offers in the early utterance time-window. In contrast to our expectations, we did not find any differences between the conditions in the lower frequencies, including the alpha/beta band (*p* > 0.43).

**FIGURE 3:**
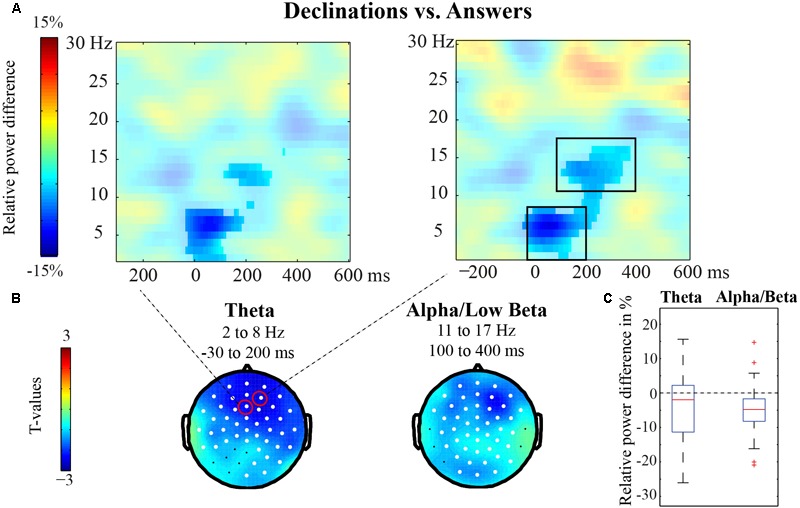
Early utterance time-window: Declinations vs. Answers. **(A)** Relative power differences between Declinations and Answers (in transparent colors) at two representative frontal sites, with the significant cluster overlaid in opaque colors. For location of the sites, see circles in **(B)**. **(B)** Topography of the effects (in *t*-values), with channels that showed a significant difference between the conditions highlighted in white. **(C)** Box-plots showing the spread of the relative power differences over participants in the right electrode displayed in **(A)** and the time- and frequency-ranges indicated with black boxes in **(A)** and with text in **(B)**.

Finally, we investigated oscillatory power differences between Declinations and Answers in the early-utterance time-window. The cluster-based permutation test for the low frequencies revealed one significant cluster comparing the two conditions (*p* = 0.02). **Figure 3** shows the relative power differences between Declinations and Answers (divided by their sum) at two representative channels with the significant cluster overlaid in opaque colors. Lower power was observed in Declinations relative to Answers mainly in the theta range (2–8 Hz) approximately from -30 to 200 ms and in the alpha/low beta range (11–17 Hz) from 100 to 400 ms. The difference in alpha/beta power between these two conditions was unexpected. Note that power differences in the alpha/beta band were not found for this late time-window in the cluster-based ANOVA with three conditions, so this effect should be interpreted with caution. The analyses with subtracted ERPs revealed one negative cluster (*p* = 0.013; see Supplementary Figure 3), reflecting a similar theta effect plus an earlier alpha/low beta effect from around -200 to 0 ms with an anterior distribution. This early alpha/low beta effect corresponds to the differences observed in the cluster-based ANOVA. As for theta, the effect found in the main analysis was confirmed in the separate theta analysis with 600 ms windows (one negative cluster, 2–5 Hz, -100 to 150 ms, *p* = 0.02). **Figure 3B** shows the topography of the effects found in the main analysis (in *t*-values, with channels that show a significant difference in the relevant time-frequency range in white). The effects were widespread over bilateral anterior sites in the theta frequencies, while the alpha/low beta was also anterior but more central (see **Figure 3B**).

For completeness, relative power differences observed in the main analysis for low frequencies at the first word are shown for all electrodes in Supplementary Figures 4–6.

In the cluster-based ANOVA approach comparing the three conditions in the gamma band (>30 Hz), we found no significant clusters (*p* > 0.7), nor in any of the pairwise comparisons between two conditions (*p*s > 0.14).

#### Final Word Time-Window

We hypothesized that Pre-offers would be associated with a power modulation in the gamma band (>30 Hz) at the utterance-final word, relative to Answers and Declinations. However, the cluster-based permutation tests in the final word time-window (from 0 to 1000 ms after final word onset) did not reveal any significant differences in the gamma frequencies, neither in the ANOVA test with three conditions (*p* > 0.7), nor in the pairwise comparisons between any of the conditions (*p* > 0.18). We present figures for the gamma range results that are relevant to our hypothesis in Supplementary Figures 7–9 show non-significant relative power differences between the speech acts in the gamma range 30–90 Hz at the utterance-final word.

**FIGURE 4:**
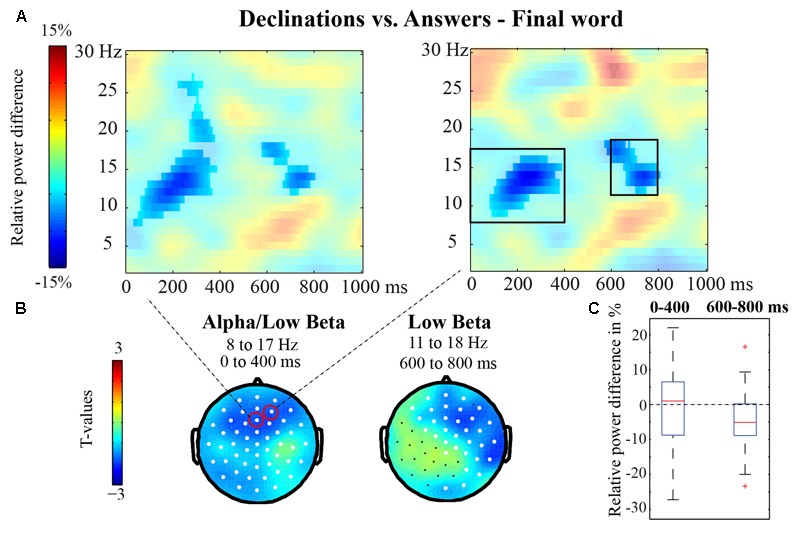
Final word time-window: Declinations vs. Answers. **(A)** Relative power differences between Declinations and Answers (in transparent colors) at two representative frontal sites, with the significant cluster overlaid in opaque colors. For location of the sites, see circles in **(B)**. **(B)** Topography of the effects (in *t*-values), with channels that showed a significant difference between the conditions highlighted in white. **(C)** Box-plots showing the spread of the relative power differences over participants in the right electrode displayed in **(A)** and the time- and frequency-ranges indicated with black boxes in **(A)** and with text in **(B)**.

In the low frequency analyses (2–30 Hz) comparing the three conditions in a cluster-based ANOVA approach, we found one significant *F*-cluster (*p* = 0.004; see Supplementary Figure 10). The same analysis with ERPs subtracted yielded a very similar cluster (*p* = 0.01). In the pairwise comparisons between two conditions, we found one negative cluster with similar characteristics as in the ANOVA analysis, reflecting lower power for Declinations relative to Answers (*p* = 0.002, see **Figure 4**) in the alpha/low beta range approximately from 0 to 400 ms (8–17 Hz) and from 600 to 800 ms (11–18 Hz). Both effects were predominantly anterior, but the earlier effect appeared more left-lateralized and the later effect more right-lateralized (see **Figure 4B**). These results were confirmed in the analyses with average ERPs subtracted (one negative cluster with *p* = 0.002; see Supplementary Figure 11). Declinations relative to Pre-offers yielded a marginally significant cluster (*p* = 0.034) with lower power for Declinations. Finally, Pre-offers vs. Answers yielded no significant clusters in the low frequency analyses (*p* > 0.3).

For completeness, relative power differences observed in the main analysis for low frequencies at the final word are shown for all electrodes in Supplementary Figures 12–14.

## Discussion

Our results show that brain oscillations differentiate speech acts during comprehension of spoken dialog. As expected, the speech act manipulation elicited early (pre-target) modulations of EEG power in the alpha/beta band, suggesting a role for anticipatory processes in speech act recognition. Additionally, we observed effects in the theta band during the beginning of the target speech acts, although we believe they have to be treated with caution. Below we discuss the study findings and implications in more detail.

### Alpha/Beta Power and Anticipation to Upcoming Speech Acts

We observed reduced power in the alpha and beta range in Declinations roughly from -200 to 0 ms before target utterance onset relative to Pre-offers and from 100 to 400 ms relative to Answers. The analyses with average ERPs subtracted indicate that the effect relative to Answers also occurs before target utterance onset, i.e., from -200 to 0 ms. Since evoked potentials can mask induced oscillations (Graimann and Pfurtscheller, 2006), the analysis with ERPs subtracted is likely to better capture the time-window of the alpha/beta effect. Moreover, the alpha/beta effect in the early time-window -200 to 0 ms was present in the cluster found by the ANOVA analysis including all three conditions, while the effect in the later 100–400 ms window was not. Thus, overall the results indicate lower alpha/beta power in Declinations relative to Pre-offers and Answers in a 200 ms time-window just before target utterance onset. Importantly, this effect is a true oscillatory response, given its presence in the control analyses with average ERPs removed.

The alpha/beta power difference in Declinations relative to Pre-offers is in agreement with the assumption that the Declinations occur in more constraining speech act contexts than Pre-offers and are hence more anticipatable (due to adjacency pair structure). The modulation of alpha/beta power in Declinations relative to Answers was unexpected, as Answers also occur in highly constraining speech act contexts and thus should involve the same degree of constraint as Declinations (see Schegloff, 2007 for a discussion on adjacency pairs). Moreover, the absence of significant differences in alpha/beta power in Answers relative to Pre-offers was unexpected. The pattern of results indicates that anticipatory processes are triggered to a larger degree in the Declination dialogs relative to the other actions. Below we provide two possible accounts for anticipatory processing prior to the Declination speech acts, one based on anticipatory attention and the other on predictive processing.

The relationship between anticipatory attention and alpha/beta desynchronization has been well established (see for instance Bastiaansen and Brunia, 2001; Bastiaansen et al., 2001; van Ede et al., 2011, 2014). Studies on attention typically report suppression of alpha and/or beta activity in the interval between some symbolic cue and the anticipated target, with the beta suppression sometimes extending into the stimulus period (van Ede et al., 2014). The pre-target alpha/beta modulation in the present study resembles such an anticipatory attention effect. What is it about Declinations that could trigger anticipatory attention? One factor that differentiates the Declinations from the other speech act conditions is what socio-emotional implications are involved in the speech act interaction. The context in the Answer dialogs (a question) simply calls for non-sensitive information and therefore does not have substantial implications for the participants in the dialog. The context in the Pre-offer dialogs states a problem of some kind without placing any obligations on the listener to remedy the problem. In contrast, the context in the Declination dialogs contains an offer, which can either be responded to with an acceptance – triggering future engagements with added implications – or be followed by a face-threatening rejection (Goffman, 1955; Brown and Levinson, 1988). There is therefore more at stake in the Declination dialogs. Strongly valenced input, including stimuli triggering negative or threat-related emotions, can enhance attention and thereby facilitate perception of events (Ohman et al., 2001; Dolan, 2002; Vuilleumier, 2005; Vuilleumier and Huang, 2009). The alpha/beta effect may reflect modulation of attention in anticipation of a reply that has the potential to be a socially dispreferred rejection. This account can explain the modulation of alpha/beta power in Declinations relative to Answers as well as the absence of alpha/beta differences in Answers relative to Pre-offers.

An alternative interpretation of the findings is that the early alpha/beta effect reflects anticipation in the form of precise predictions. While participants in the current study could not use statistical regularities in the experimental paradigm to make predictions about the target speech act, due to additional filler dialogs, it is possible that the alpha/beta effect reflects expectations based on prior knowledge. Alpha/beta oscillations in the pre-stimulus interval have been found to be modulated by the predictability of the upcoming stimulus event, even in the absence of attention (van Ede et al., 2010, 2014; Bauer et al., 2014; Todorovic et al., 2015). For instance, expectations regarding the onset of a tone stimulus lead to a pre-stimulus decrease in beta power when it is unattended (Todorovic et al., 2015). In the case of the Declination dialogs, the context (an offer) should be followed by an acceptance or declination, but acceptances are likely more common in conversation. The pre-stimulus alpha/beta effect may reflect the prediction that an acceptance is underway, a prediction which is then disconfirmed when the incoming target utterance makes clear that a prototypical acceptance is not in progress. Such a prediction is not possible in the Pre-offer dialogs, since the context utterance can be followed by a large number of actions. In the Answer dialogs participants can predict that an answering action will follow the context (a question), but this is rather trivial in comparison to the content of the answer (e.g., “a credit-card,” “cash,” etc.), which cannot be predicted. Thus the context utterance could enable more pre-target predictive processing in the Declination dialogs relative to Pre-offers and Answers.

At present we cannot determine the relative likelihood of these two scenarios, i.e., anticipation based on anticipatory attention or predictive processing. Although attention vs. expectation or prediction are often thought of as a single mechanism that facilitates recognition, they operate in different ways (Arnal and Giraud, 2012; see also Todorovic et al., 2015). While expectation leads to reduced neural responses to expected events (post-stimulus), attention increases the response to expected/attended events (for a discussion, see Arnal and Giraud, 2012; Todorovic et al., 2015). However, it is difficult to disentangle effects of attention and prediction/expectation and these processes interact during sensory processing (Todorovic et al., 2015).

In addition to alpha/beta power decrease around the start of the target utterance in Declinations, we also observed power modulations in these frequency bands in Declinations relative to Answers at the utterance-final word. A recent study on sentence processing reported a decrease in alpha/beta power for late words in a sentence in comparison to early words (Lam et al., 2016). This was interpreted as a facilitatory effect of context on semantic and syntactic unification. Similarly, the context in the Declinations dialogs could have a facilitatory effect on linguistic processing at the utterance-final word. The first two words of the target utterance (*I have…*) make clear that a proto-typical acceptance is not underway, strengthening the likelihood that a declination is involved and hence facilitating processing of the final word. Alternatively, the social relevance of the Declination speech acts could call for increased attention throughout the utterance. All in all, the final-word alpha/beta decrease in Declinations relative to Answers is in line with research showing alpha/beta power modulations during sentence endings in highly constraining contexts.

The critical finding from this study is that anticipatory processes are involved in speech act recognition and that they are dependent on the speech act type. The early alpha/beta effect in Declinations relative to Answers and Pre-offers converges with results from a previous ERP study supporting early speech act recognition in Declinations (Gisladottir et al., 2015). The results go beyond prior research on EEG signatures of anticipation by showing that anticipatory alpha/beta can be induced by the pragmatics of speech acts.

### Theta Power Modulations

We observed unexpected differences between the three speech act conditions in the theta band. Less power was found in the theta range from approximately -30 to 200 ms after target utterance onset in Declinations relative to Answers and Pre-offers. However, the effect was not present in the additional theta analyses (with larger time-windows) when comparing all three conditions and in the pairwise comparison between Declinations and Pre-offers. Thus, these effects should be treated with caution and future research is necessary to replicate them. Unlike the alpha and beta bands, synchronization in theta frequencies is generally thought to reflect active processing, while desynchronization reflects deactivation. For instance, theta power increases with working memory load (Jensen and Tesche, 2002) and top-down cognitive processing (Min and Park, 2010). In language research, increased theta power has been associated with the retrieval of lexical-semantic information and larger demands on verbal working memory (Bastiaansen et al., 2008; Spotorno et al., 2013; Lam et al., 2016). The lexical-semantic and memory accounts imply that there should be decreased processing in Declinations relative to Answers, which is contrary to the assumptions that Answers and Declinations are both relatively anticipatable and that anticipatory attention increases the response to incoming stimuli (as discussed in section “Alpha/Beta Power and Anticipation to Upcoming Speech Acts”). A lexical-semantic or memory explanation is also contrary to the predictive account above, since a failed prediction should lead to increased processing during the beginning of Declinations relative to Answers when listeners detect that the incoming utterance does not match the prediction for an Acceptance (see, for instance, Dikker and Pylkkänen, 2013). However, theta has also been shown to play an inhibitory role in language regions (Hermes et al., 2014). Theta power is decreased during a verb generation task and correlates negatively with fMRI BOLD, indicating that higher amplitude theta results in low metabolic expenditure (Hermes et al., 2014). Under such an inhibitory account, lower theta power could reflect increased activation in Declinations relative to Answers, for instance in language regions. This would be in line with the interpretation that a failed prediction leads to increased processing effort when the mismatch is recognized. While the functional significance of the theta effect in the present study remains unclear, our results add to the growing body of research indicating that theta plays a role in the processing of linguistic input.

### Absence of Effects in the Gamma Frequency Range

Contrary to our expectations, we did not observe any oscillatory power differences between Pre-offers and the other speech acts (Answers, Declinations) in the gamma range at the final word. In comparison to Declinations and Answers, Pre-offers occur in less-constraining contexts and invite more inferences about upcoming talk (i.e., that a more direct offer is underway). The prior study by Gisladottir et al. (2015) reported a late negativity at the utterance-final word in Pre-offers, which was taken to reflect this complexity. In the current study we hypothesized that the oscillatory EEG signal would show evidence of speech act recognition only late in the utterance for this speech act type. Based on prior studies in the field of linguistic pragmatics (Hagoort et al., 2004; Spotorno et al., 2013) we speculated that this would be reflected in modulations of gamma oscillations at the final word relative to the other conditions. The fact that no final-word effects were observed in Pre-offers relative to Answers and Declinations suggests that ERPs are more sensitive than oscillations to the aspect of speech act comprehension reflected by the late negativity. However, prior studies reporting gamma oscillations to pragmatic phenomena have investigated irony (Spotorno et al., 2013) and world knowledge violations (Hagoort et al., 2004), which may involve bigger differences between targets and control conditions than the everyday speech acts presented in the current study. Moreover, caution must be taken when analyzing and interpreting neuronal gamma-band activity at the sensor level since cranial muscle activity can contaminate EEG recordings, with the consequence that effects are missed or misinterpreted (Whitham et al., 2007; Hipp and Siegel, 2013). Thus, although the cluster-based permutation tests for the gamma band were not significant (*p* > 0.7) we cannot rule out that speech act comprehension involves power modulations in the gamma frequency range. Readers can refer to Supplementary Material for figures of the gamma range results relevant to our hypothesis at the utterance-final word (Supplementary Figures 4–6).

### Task Demands

The current investigation employed an overhearing paradigm in which participants listened to short, pre-designed spoken dialogs and answered comprehension questions about them. The behavioral results show that while overall accuracy in the task was high (92.7%), accuracy was lower in Pre-offers (85.2%) than in Answers (97.4%) and Declinations (95.5%). Lower accuracy in Pre-offers is consistent with the assumption that they occur in less constraining contexts and may therefore be less readily recognized than the other two speech acts. This pattern of behavioral results was not found in the prior ERP study on the same dialogs (Gisladottir et al., 2015), which reported considerably higher accuracy for Pre-offers (94.8%). The ERP study used a different task (speech act categorization) and did not include filler dialogs. This raises the question how the experimental paradigm influenced the current behavioral and electrophysiological results. It is possible that when participants have less top-down information about the target speech acts – due to more minimal task demands and distracting fillers – participants take a wait-and-see approach for the Pre-offers, resulting in lower accuracy. This could also help explain the absence of late gamma effects in Pre-offers, discussed in Section “Absence of Effects in the Gamma Frequency Range.”

## Conclusion

How is it that listeners can recognize speech acts so efficiently, evidenced by the extraordinarily fast transitions between turns in conversation? The primary goal of this study was to determine the time-course of speech act recognition by investigating oscillatory activity during comprehension of spoken dialog. We observed reduced power in the alpha/beta bands just prior to Declination speech acts relative to Answers and Pre-offers. The early alpha/beta effect in Declinations relative to Answers and Pre-offers converges with results from a previous ERP study supporting early speech act recognition in Declinations (Gisladottir et al., 2015). Based on the association of alpha/beta desynchronization with anticipation, the current results are taken to indicate the involvement of anticipatory processes in early speech act recognition. Anticipation of speech acts could be critical for efficient turn-taking, allowing listeners to quickly extract speech acts from the linguistic code and respond within the 200 ms time frame characteristic of conversation (Levinson, 2013; Levinson and Torreira, 2015). The results show that anticipatory processes are dependent on the characteristics of the interaction, including the speech act type.

## Author Contributions

RG designed the experiment. SL developed the theoretical framework. RG carried out the experiment. SB and RG analyzed the data and wrote the manuscript.

## Conflict of Interest Statement

The authors declare that the research was conducted in the absence of any commercial or financial relationships that could be construed as a potential conflict of interest.

## Footnotes

1 The artifact rejection was based on baselined data, using a 150 ms baseline prior to the gap between the two utterances, but non-baselined data were used for the time-frequency analysis.

2 Time-frequency analysis using Hanning window, multitapers and wavelets (2014). Fieldtriptoolbox.org. Available at: http://fieldtrip.fcdonders.nl/tutorial/timefrequencyanalysis [accessed May 22, 2017].
