# Supplementary material for: Oscillatory Brain Responses Reflect Anticipation during Comprehension of Speech Acts in Spoken Dialog
Source: Front Hum Neurosci. 2018 Feb 7;12:34. doi: 10.3389/fnhum.2018.00034 (PMC5808328; doi:10.3389/fnhum.2018.00034)
Supplement: Supplementary file 1 [file Data_Sheet_1.pdf]

Supplementary Material

Oscillatory brain responses reflect anticipation during comprehension of speech acts in spoken dialog

Rosa S. Gisladottir*, Sara Bögels and Stephen C. Levinson

*** Correspondence:** Rosa S. Gisladottir: rosasigny@gmail.com

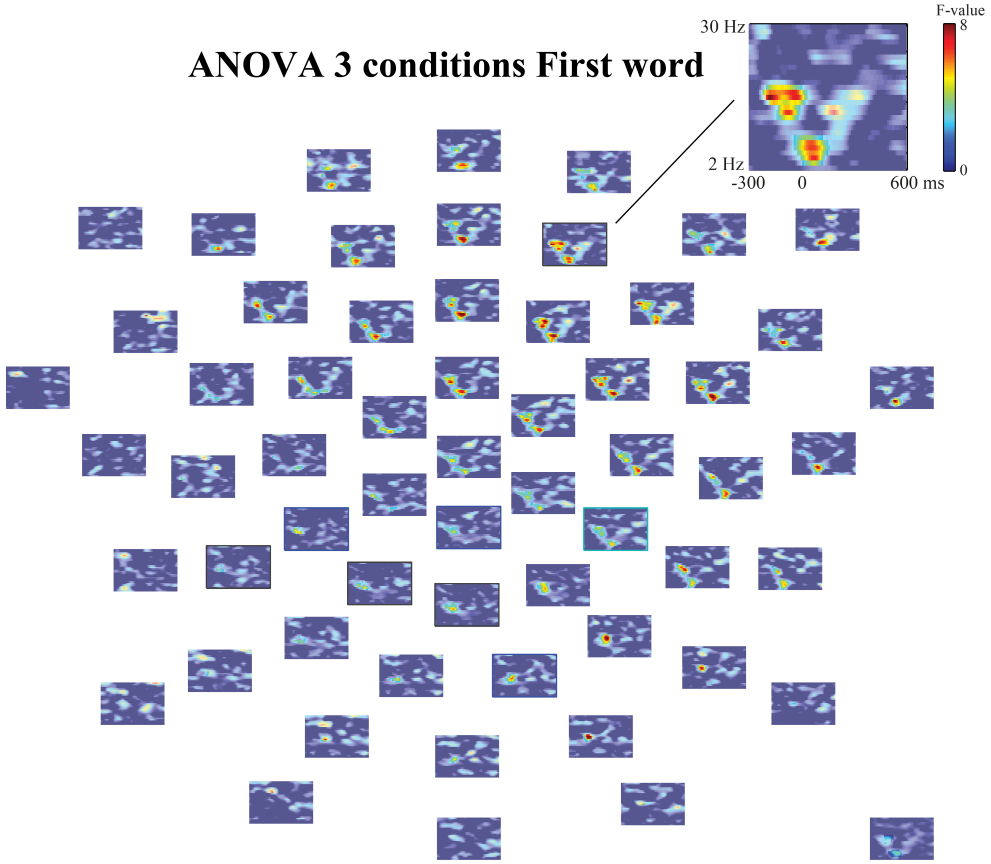

Supplementary Figure 1. *F*-values for the cluster-based ANOVA analyses including all three speech act conditions, shown for all electrodes in the low-frequency range (2-30 Hz) at the first word (-300 to 600 ms). *F*-values are presented in transparent colours with the marginally significant clusters overlaid in opaque colours.

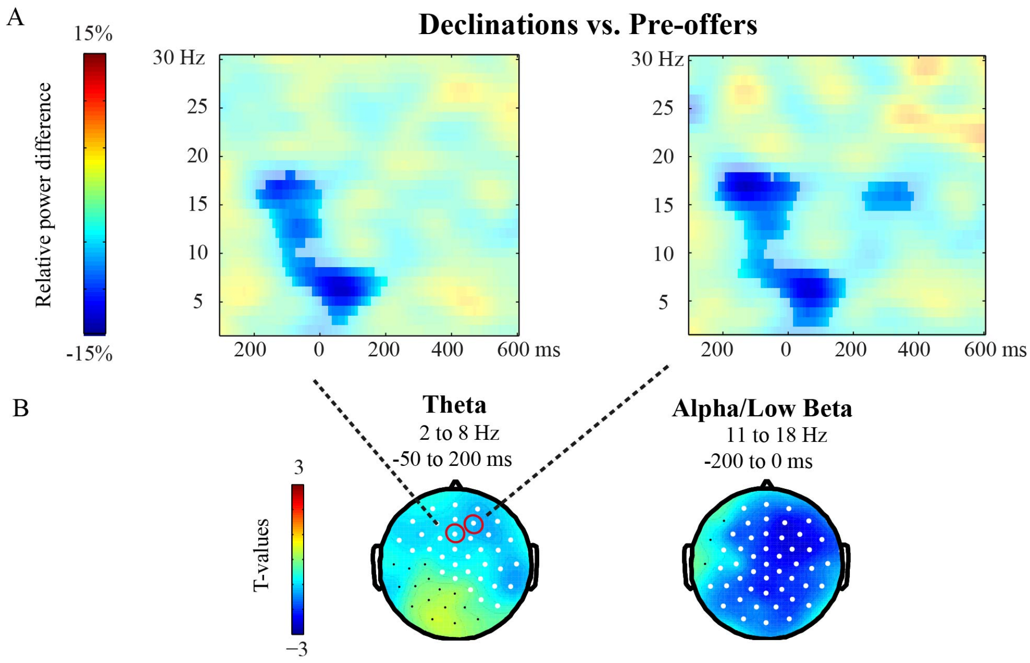

Supplementary Figure 2. Analyses with subtraction of average ERP per participant. Early utterance time-window: Declinations vs. Pre-offers. A) Relative power differences between Declinations and Pre-offers (in transparent colours) at two representative frontal sites with the significant cluster overlaid in opaque colours. For location of the sites, see circles in panel B. B) Topography of the effects (in t-values), with channels that showed a significant difference between the conditions highlighted in white.

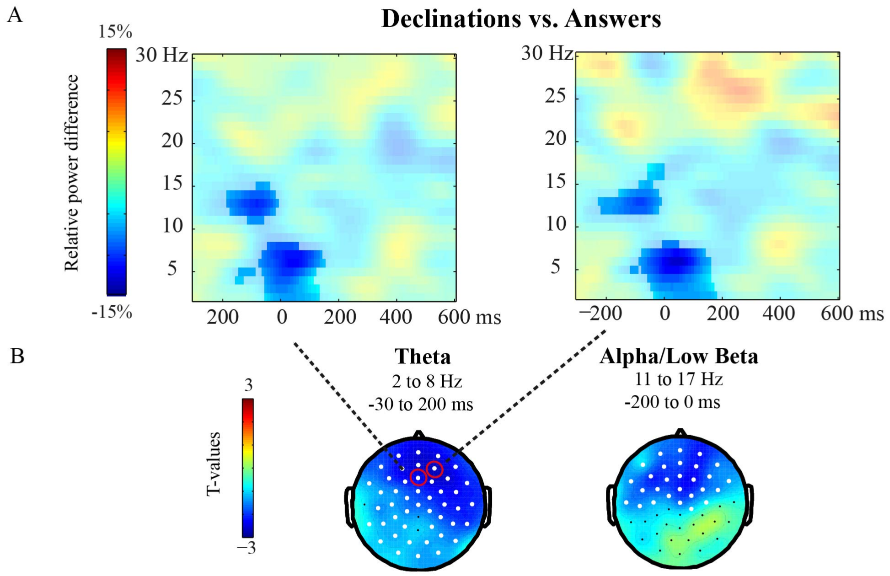

Supplementary Figure 3. Analyses with subtraction of average ERP per participant. Early utterance time-window: Declinations vs. Answers. A) Relative power differences between Declinations and Answers (in transparent colours) at two representative frontal sites, with the significant cluster overlaid in opaque colours. For location of the sites, see circles in panel B. B) Topography of the effects (in t-values), with channels that showed a significant difference between the conditions highlighted in white.

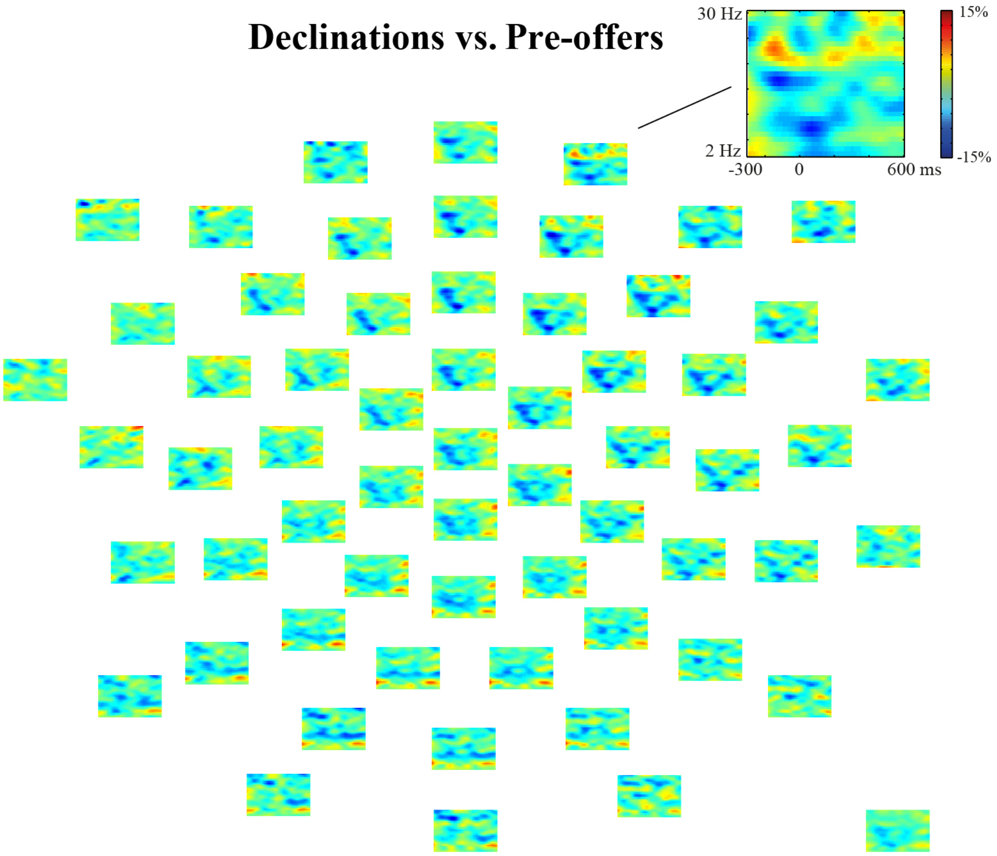

Supplementary Figure 4. Relative power differences shown for all electrodes in the low-frequency range (2-30 Hz) at the first word (-300 to 600 ms) for Declinations vs. Pre-offers.

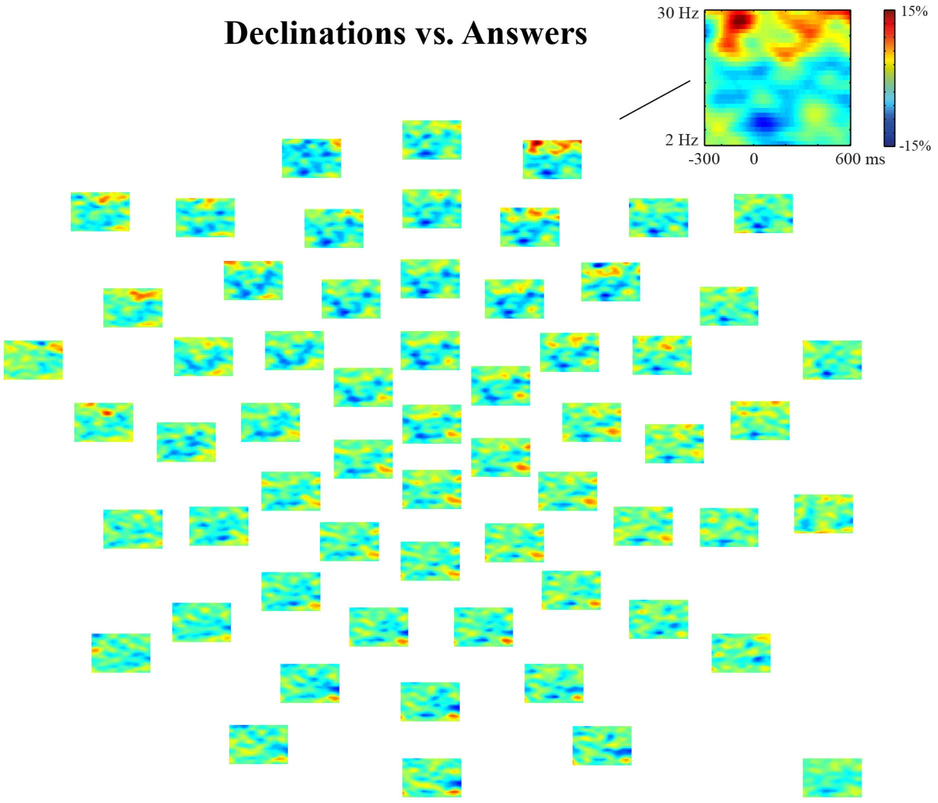

Supplementary Figure 5. Relative power differences shown for all electrodes in the low-frequency range (2-30 Hz) at the first word (-300 to 600 ms) for Declinations vs. Answers.

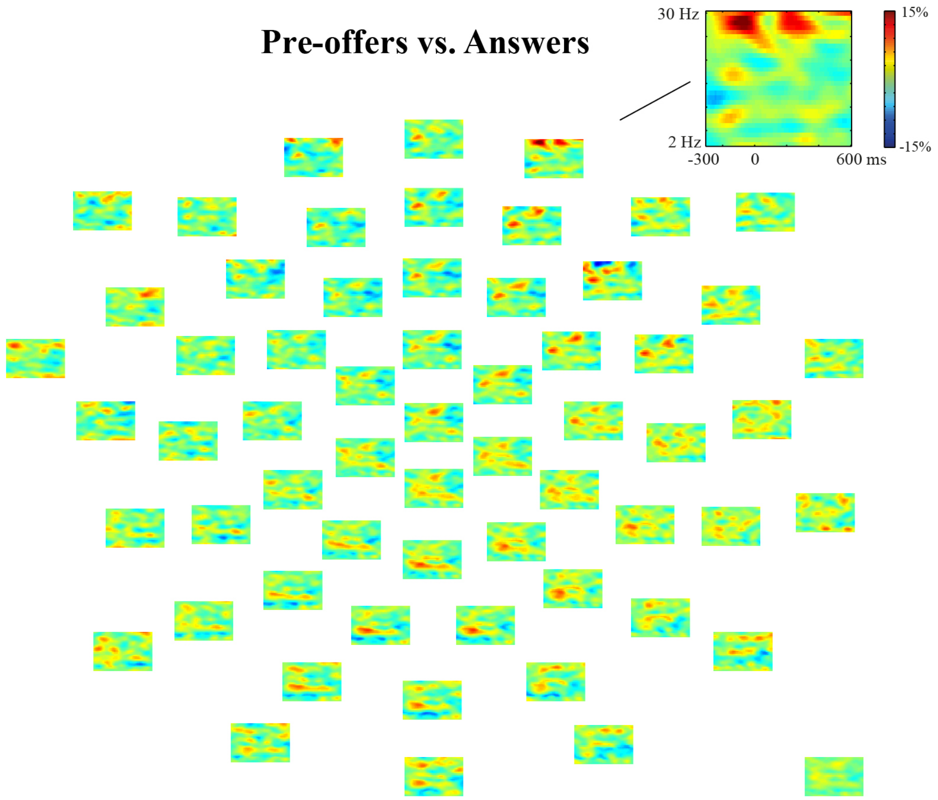

Supplementary Figure 6. Relative power differences shown for all electrodes in the low-frequency range (2-30 Hz) at the first word (-300 to 600 ms) for Pre-offers vs. Answers.

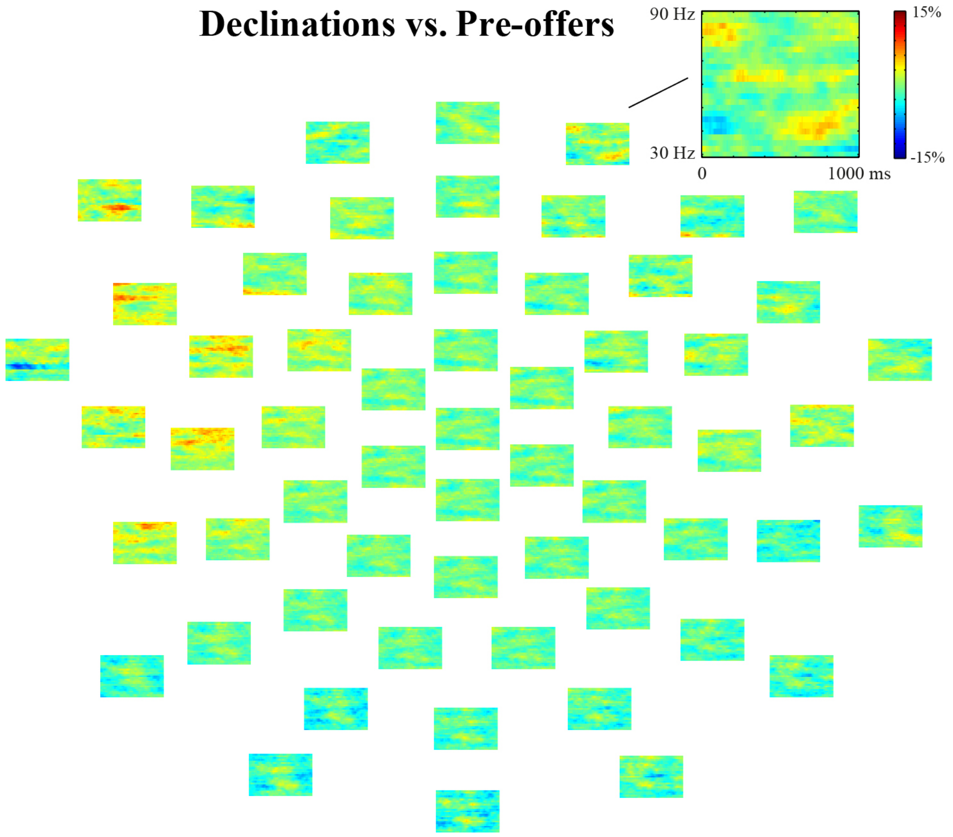

Supplementary Figure 7 Relative power difference in the gamma range (30-90 Hz) at the final word (0-1000 ms) for Declinations vs. Pre-offers.

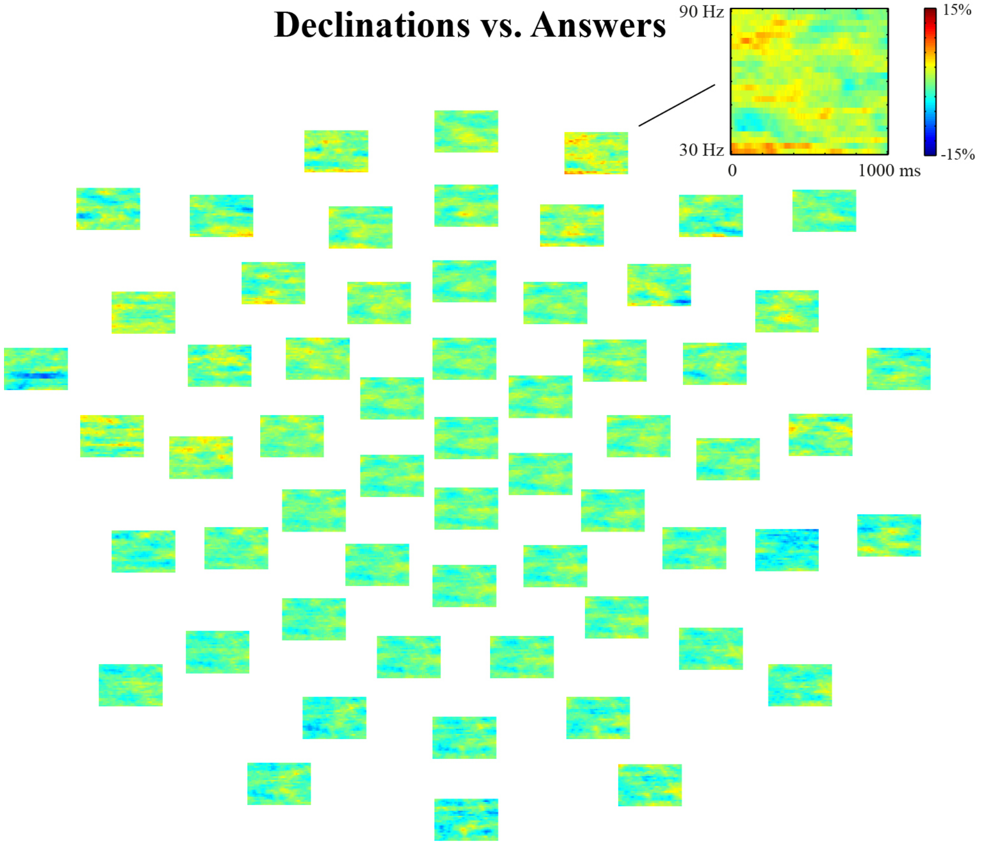

Supplementary Figure 8. Relative power difference in the gamma range (30-90 Hz) at the final word (0-1000 ms) for Declinations vs. Answers.

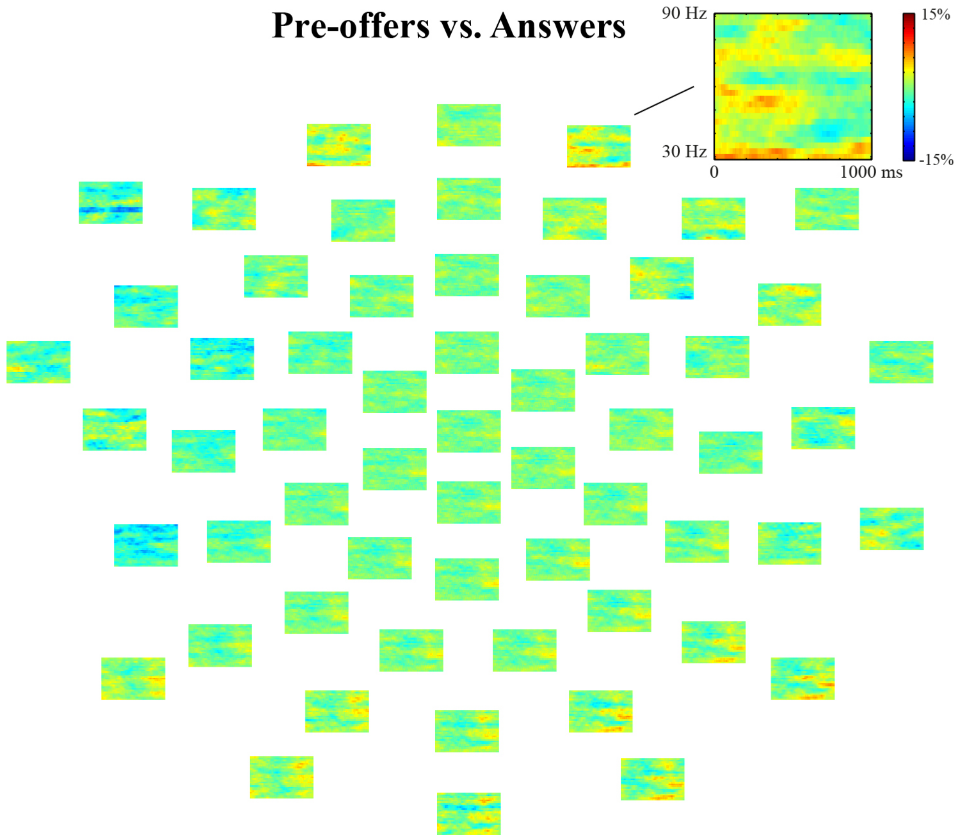

Supplementary Figure 9. Relative power difference in the gamma range (30-90 Hz) at the final word (0-1000 ms) for Pre-offers vs. Answers.

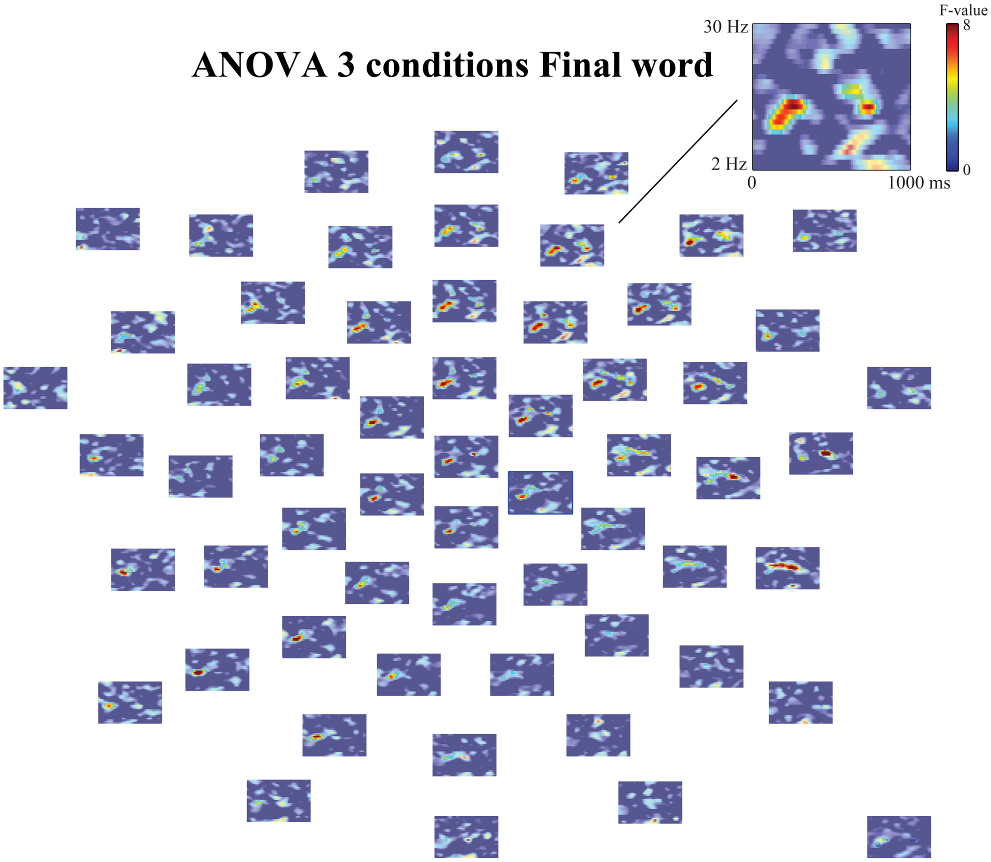

Supplementary Figure 10. *F*-values for the cluster-based ANOVA analyses including all three speech act conditions, shown for all electrodes in the low-frequency range (2-30 Hz) at the utterance-final word (0 to 1000 ms). *F*-values are presented in transparent colours with the significant cluster overlaid in opaque colours.

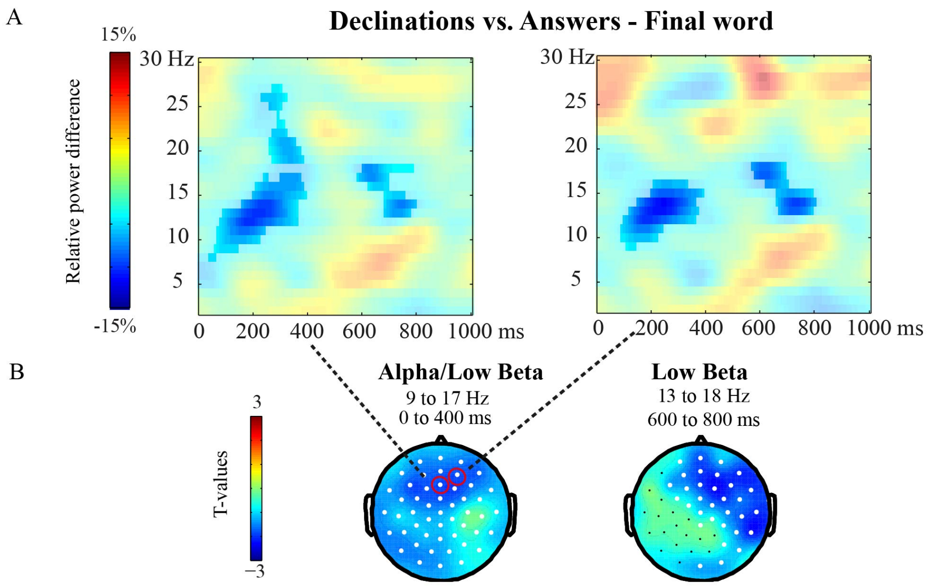

Supplementary Figure 11. Analyses with subtraction of average ERP per participant. Final word time-window: Declinations vs. Answers. A) Relative power differences between Declinations and Answers (in transparent colours) at two representative frontal sites, with the significant cluster overlaid in opaque colours. For location of the sites, see circles in panel B. B) Topography of the effects (in t-values), with channels that showed a significant difference between the conditions highlighted in white.

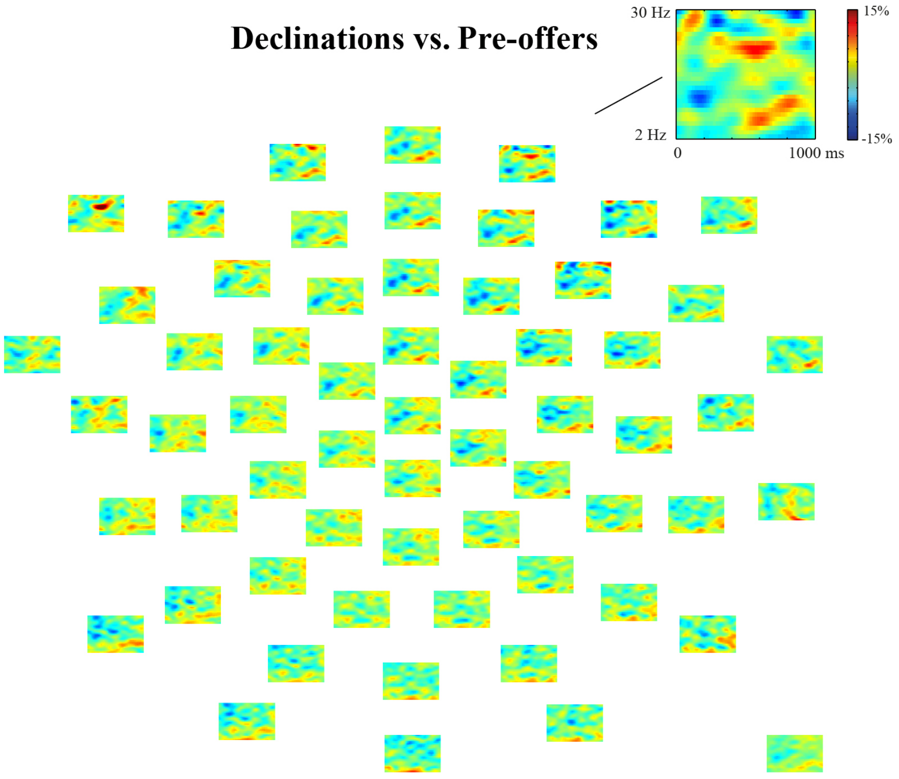

Supplementary Figure 12. Relative power differences shown for all electrodes in the low-frequency range (2-30 Hz) at the final word (0-1000 ms) for Declinations vs. Pre-offers.

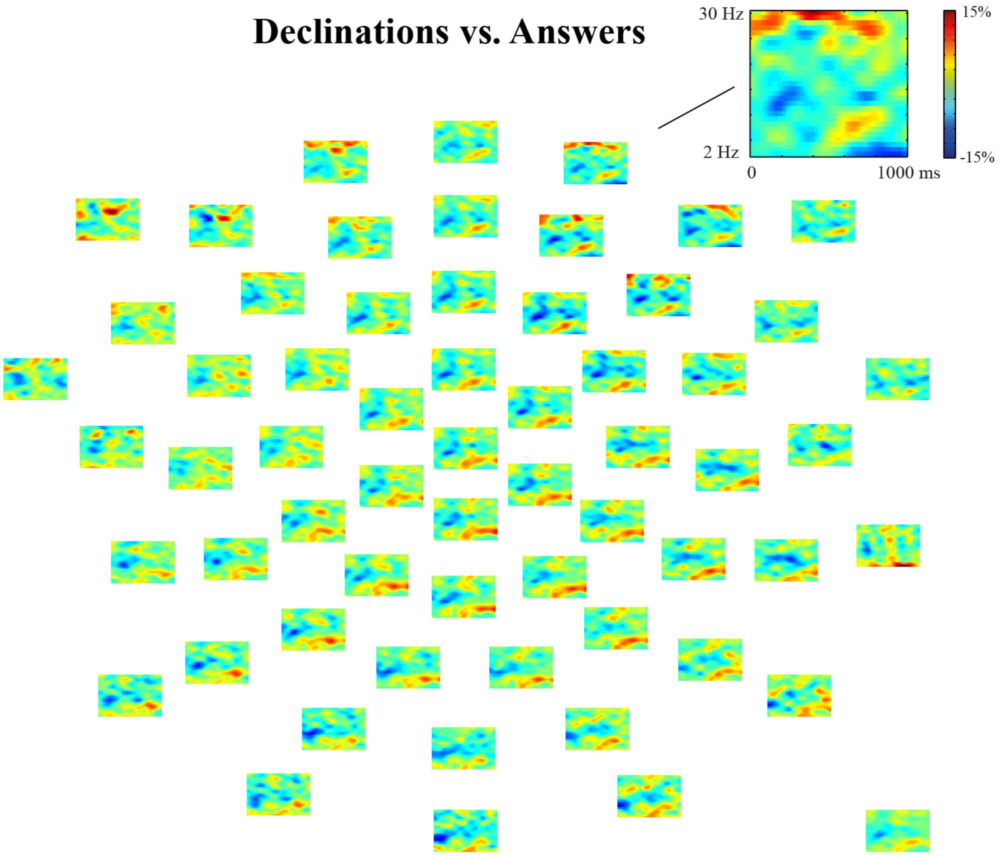

Supplementary Figure 13. Relative power differences shown for all electrodes in the low-frequency range (2-30 Hz) at the final word (0-1000 ms) for Declinations vs. Answers.

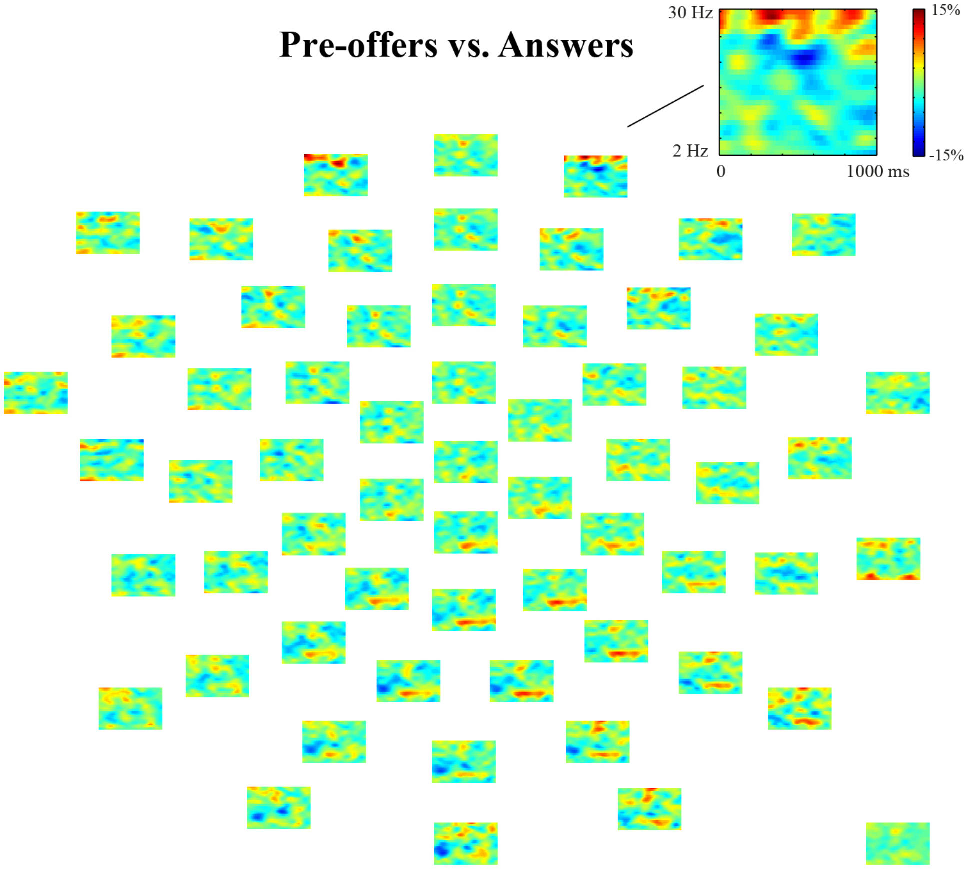

Supplementary Figure 14. Relative power differences shown for all electrodes in the low-frequency range (2-30 Hz) at the final word (0-1000 ms) for Pre-offers vs. Answers.
